# Saline-alkali stress affects the accumulation of proanthocyanidins and sesquiterpenoids via the MYB5-ANR/TPS31 cascades in the rose petals

**DOI:** 10.1093/hr/uhae243

**Published:** 2024-08-30

**Authors:** Qiao Wang, Baoquan Du, Yujing Bai, Yan Chen, Feng Li, Jinzhe Du, Xiuwen Wu, Liping Yan, Yue Bai, Guohua Chai

**Affiliations:** College of Resources and Environment, Qingdao Agricultural University, No. 700 Changcheng Road, Chengyang District, Qingdao 266109, China; Academy of Dongying Efficient Agricultural Technology and Industry on Saline and Alkaline Land in Collaboration with Qingdao Agricultural University, No. 7 Zhihui Road, Guangrao County, Dongying 257000, China; College of Resources and Environment, Qingdao Agricultural University, No. 700 Changcheng Road, Chengyang District, Qingdao 266109, China; College of Resources and Environment, Qingdao Agricultural University, No. 700 Changcheng Road, Chengyang District, Qingdao 266109, China; Academy of Dongying Efficient Agricultural Technology and Industry on Saline and Alkaline Land in Collaboration with Qingdao Agricultural University, No. 7 Zhihui Road, Guangrao County, Dongying 257000, China; College of Landscape Architecture and Forestry, Qingdao Agricultural University, No. 700 Changcheng Road, Chengyang District, Qingdao 266109, China; Forestry College, Inner Mongolia Agricultural University, No. 306 Zhaowuda Road, Saihan District, Huhhot 010018, China; Academy of Dongying Efficient Agricultural Technology and Industry on Saline and Alkaline Land in Collaboration with Qingdao Agricultural University, No. 7 Zhihui Road, Guangrao County, Dongying 257000, China; College of Landscape Architecture and Forestry, Qingdao Agricultural University, No. 700 Changcheng Road, Chengyang District, Qingdao 266109, China; Academy of Dongying Efficient Agricultural Technology and Industry on Saline and Alkaline Land in Collaboration with Qingdao Agricultural University, No. 7 Zhihui Road, Guangrao County, Dongying 257000, China; College of Agronomy, Qingdao Agricultural University, No. 700 Changcheng Road, Chengyang District, Qingdao 266109, China; College of Resources and Environment, Qingdao Agricultural University, No. 700 Changcheng Road, Chengyang District, Qingdao 266109, China; Shandong Provincial Academy of Forestry, No. 42 Wenhua Dong Road, Lixia District, Jinan 250014, China; Forestry College, Inner Mongolia Agricultural University, No. 306 Zhaowuda Road, Saihan District, Huhhot 010018, China; College of Resources and Environment, Qingdao Agricultural University, No. 700 Changcheng Road, Chengyang District, Qingdao 266109, China; Academy of Dongying Efficient Agricultural Technology and Industry on Saline and Alkaline Land in Collaboration with Qingdao Agricultural University, No. 7 Zhihui Road, Guangrao County, Dongying 257000, China

## Abstract

Rose (*Rosa rugosa*) petals are rich in diverse secondary metabolites, which have important physiological functions as well as great economic values. Currently, it remains unclear how saline and/or alkaline stress(es) influence the accumulation of secondary metabolites in rose. In this study, we analyzed the transcriptome and metabolite profiles of rose petals under aline–alkali stress and uncovered the induction mechanism underlying major metabolites. Dramatic changes were observed in the expression of 1363 genes and the abundances of 196 metabolites in petals in response to saline–alkali stress. These differentially expressed genes (DEGs) and differentially accumulated metabolites (DAMs) are mainly associated with flavonoid and terpenoid metabolism and the reconstruction of cell walls. Of them, *TERPENE SYNTHASE 31* (*TPS31*) overexpression in tobacco leaves driven by its own promoter resulted in significant alterations in the levels of diverse terpenoids, which were differentially influenced by saline–alkali stress. An integrated analysis of metabolomic and transcriptomic data revealed a high correlation between the abundances of flavonoids/terpenoids and the expression of the transcription factor MYB5. MYB5 may orchestrate the biosynthesis of sesquiterpenoids and proanthocyanidins through direct regulation of *TPS31* and *ANR* expression under aline–alkali stress. Our finding facilitates improving the bioactive substance accumulation of rose petals by metabolic engineering.

## Introduction

Soil salinize–alkalization is a worldwide problem. At present, there are ~8.31 × 10^8^ ha of land suffering from varying degrees of salinization and alkalinization in the world, and this area continues to increase [1]. To survive in saline–alkali soils, plants have evolved various adaptive mechanisms, including gene expression regulation, protein synthesis and turnover, and carbohydrate and energy metabolism [2]. Compared with well-studied Arabidopsis and agricultural crops [3, 4], the adaptable mechanism of perennial trees under high salinity and alkalinity remains unclear.

Rose (*Rosa rugosa*) is a stress-tolerant shrub that originates from China. The rose flowers are rich in a variety of compounds, including primary metabolites (e.g. amino acids and polysaccharides) and secondary metabolites (e.g. flavonoids, terpenoids, and polyphenolics) [5]. These metabolites have broad applications in the cosmetic, medicine, and food industries. Biosynthesis and regulation of these metabolites are drastically influenced by environmental factors including salinity and alkalinity, and many of them help plants to cope with the negative effects of these stresses [6]. Elucidating the biosynthetic mechanisms of major metabolites in rose is helpful for accelerating microbial synthesis of these natural products.

Flavonoids and terpenoids represent the largest group of natural products in plants and have critical physiological and metabolic functions [7]. Flavonoids consist of a large group of phenylpropanoid compounds that includes flavanones, flavones, flavanols, anthocyanidins, and proanthocyanins (PAs), according to the modification of their basic structure. In rose, the structural gene *ANTHOCYANIDIN REDUCTASE* (*ANR*) of the flavonoid pathway is responsive for biosynthesis of proanthocyanidins in flowers and promotes plant resistance against oxidative stress in transgenic tobacco [8]. Terpenoids mainly include monoterpenoids, diterpenoids, sesquiterpenoids, triterpenoids, and their derivatives. Biosynthesis of terpenoids requires two independent and compartmentally separated pathways: the MEVALONIC ACID (MVA) and METHYLERYTHRITOL PHOSPHATE (MEP) pathways [9]. In rose, the structural gene *FARNESYL DIPHOSPHATE SYNTHASE 1* (*FPPS1*) of the terpenoid pathway catalyzes the formation of FARNESYL DIPHOSPHATE (FPP), which provides a precursor for sesquiterpene and triterpene biosynthesis, and is upregulated by salt treatment [10].

Regulation of flavonoid and terpenoid biosynthesis by saline and/or alkaline stresses predominantly occurs at the transcriptional level, involving multiple transcription factors (TFs) families (MYB, bHLH, WRKY, bZIP, AP2/ERF, and NAC) [11, 12]. For instance, different sorghum MYB members induce and suppress some flavonoid structural genes to facilitate the balance of flavonoid levels under saline–alkali stress [13]. In fact, the use of a single TF to control both flavonoid and terpenoid pathways is difficult. Currently, only several such MYBs have been identified to control both flavonoid and terpenoid metabolism. In white grapeberries, MYB24 regulates drought-induced terpene and flavonol synthesis [14]. In variegated grape berries, MYB24 orchestrates flavonol and monoterpene metabolism as light responses to anthocyanin depletion [15]. In tomato, MYB75 positively regulates the synthesis of anthocyanin and aroma volatile, but negatively regulates the accumulation of sesquiterpenes [16, 17]. In rose, MYB5 regulates the anthocyanin and proanthocyanidin synthesis in feedback loop responding to wounding and oxidation [18]. MYB23, the homolog of MYB5 in *Artemisia annua*, acts a positive regulator of trichome development and artemisinin synthesis [19]. To date, it is unknown whether and how MYB5 functions in the flavonoid and terpenoid pathways responsive to saline–alkali stress.

**Fig. 1:**
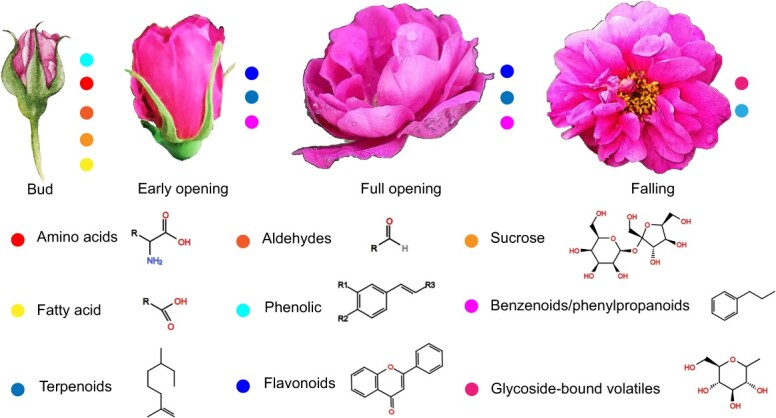
Major metabolites in the rose petals during flower development. Bud, the petals are covered by sepals; early opening, time before bloom; full opening, bloom time; falling, the petals begin to fall. Dots with different colors show major metabolites in the petals at each developmental stage.

In this study, we investigated the effects of saline–alkali stress on the accumulation of secondary metabolites in the rose petals by integration of metabolomics and transcriptomics. The abundances of flavonoids and terpenoids, the most significantly changed metabolites under stress, were highly correlated with the expression of *MYB5*. Biochemical assays were conducted to determine whether MYB5-mediated regulation of *TERPENE SYNTHASE 31* (*TPS31*) and *ANR* expression influences the sesquiterpenoid and proanthocyanidin accumulation in response to saline–alkali stress. This study provided an insight into the regulatory mechanisms of flavonoid and terpenoid accumulation in rose petals under saline–alkali stress condition.

## Results

### Major metabolites at the rose flower developmental stages

The type and content of organic compounds in flowers are tightly associated with the developmental stages [20]. To determine when the petals of rose are sampled for ‘multi-omic’ analyses, we analyzed the major metabolites in petals at different flower developmental stages. Four developmental stages containing different metabolites in the rose petals were defined (Fig. 1) based on the data of previous studies [21]. At bud stage, phenolic compounds, amino acids, aldehydes, sucrose, and fatty acid are the main components in petals. At early opening stage, pheylpropanoids/benzenoids, flavonoids, and terpenoids exhibit significant accumulation, followed by a gradual increase from early opening stage to full opening stage. Similar fragrance components were also detected at this stage in *Jasminum sambac* [22], *Borago officinalis* [23], and *Centaurea cyanus* [23]. At falling stage, the contents of terpenoids decrease and the contents of glycoside-bound volatiles remain relatively high in the rose petals (Fig. 1).

### Differential accumulation of metabolites in the rose petals under saline–alkali stress

**Fig. 2:**
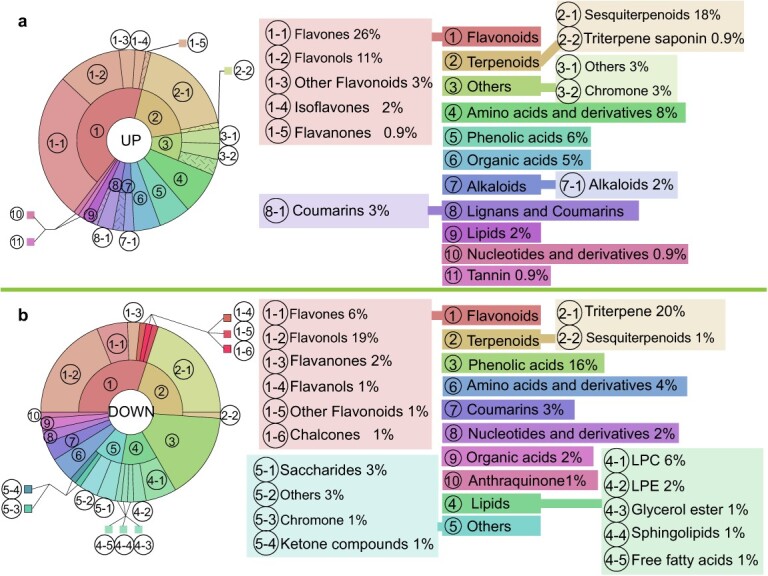
DAMs in the rose petals under saline–alkali stress. (a, b) Statistic analysis of upregulated (a) and downregulated (b) metabolites.

To investigate how saline–alkali stress influences metabolite accumulation in the rose petals, we conducted ultra-performance liquid chromatography/tandem mass spectrometry (UPLC-MS/MS) analysis of stressed petals at full opening flower stage. At this time, the petals contain the highest levels of flavonoids and terpenoids (Fig. 1). No significant morphological changes were observed between the controls and plants that were treated with the saline (0.4%)–alkali (pH = 8.0) solution for 1 week. Principal component analysis (PCA) revealed that the spatial distribution of three biological replicates for the control (CK) or treated (SA) plants was relatively close (Fig. S1a), indicating good repeatability of the metabolomic data. A total of 1815 metabolites were detected in the rose petals. Of them, the abundances of 196 metabolites showed ≥2-fold changes in SA plants compared with CK plants, with 106 metabolites being upregulated and 90 metabolites being downregulated (Table S1; Fig. S1b). As expected, flavonoids and terpenoids accounted for the largest proportions of differentially accumulated metabolites (DAMs) (Fig. 2). In upregulated metabolites, the ratios of flavonoids and terpenoids were 42.9% and 18.9%, respectively (Fig. 2a). Flavones of flavonoids, including acacetin and cirsimaritin, had the most significant changes. The largest changed terpenoids were sesquiterpenoids, including citronellol and rosacorenol. In downregulated metabolites, the ratios of flavonoids and terpenoids were 30% and 21%, respectively (Fig. 2b). The largest changed flavonoids were flavonols, including tamarixetin and patuletin. The largest changed terpenoids were triterpenes, including madasiatic acid and 2,3,23-Trihydroxyurs-12-en-28-oic acid. These results indicated that saline–alkali stress caused significant changes of diverse flavonoids and terpenoids in the rose petals at full opening stage.

**Fig. 3:**
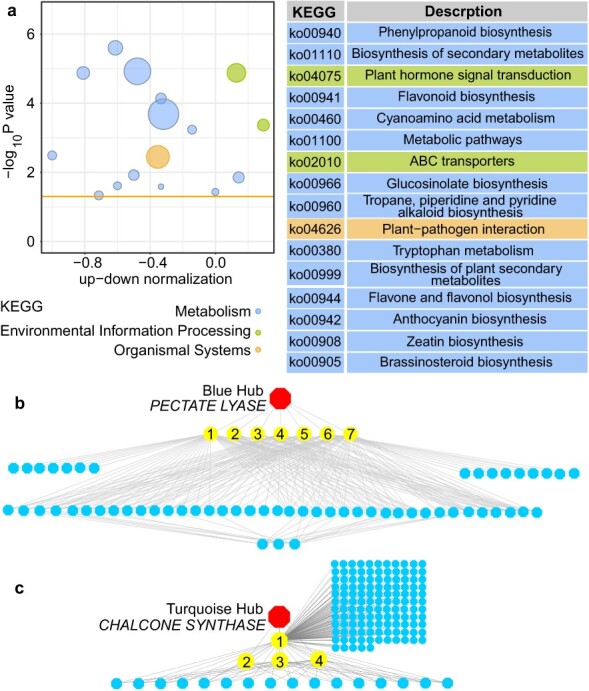
DEGs in the rose petals under saline–alkali stress. (a) KEGG pathway enrichment of DEGs. The number of DEGs is represented by the size of circle. (b, c) Gene coexpression networks with hub genes. Red dot represents a hub gene, yellow dot nodes represent genes in modules coexpressed with the hub gene, and blue dot nodes represent genes with a higher connectivity in the corresponding network. In blue module (b), 1: UDP-glucuronosyl transferase (UGT); 2: Glucosyl transferase; 3: Brassinosteroid insensitive 1 associated kinase receptor 1 (BAK1); 4: UDP-sulfoquinovose synthase; 5: Glucosyl transferase; 6: Xyloglucosyl transferase; 7: Glycosyl transferase. In turquoise module (c), 1: Aldehyde oxidase; 2: CASP-like protein; 3: Ammonium transporter; 4: Beta-amyrin 28-monooxygenase.

### Differential expression of genes in the rose petals under saline–alkali stress

To investigate how saline–alkali stress influences gene expression in the rose petals, we conducted transcriptomic analysis of the materials that were used in metabolome. A total of 76.56G clean reads were obtained from six cDNA libraries after filtering low-quality reads and adapter sequences. These six libraries were derived from untreated and treated plants with three biological replicates. PCA results showed similar expression profiles among three biological replicates for each treatment (Fig. S2a), indicating high reliability of the sequencing data. More than 85% clean reads can be mapped to the rose genomic database (http://eplantftp.njau.edu.cn/Rosa_rugosa), producing 17 735 genes. Based on the filter criteria of |log_2_ Fold Change| ≥ 1 and FDR < 0.05, a total of 1363 differentially expressed genes (DEGs) were identified, with 649 DEGs being upregulated and 714 DEGs being downregulated (Table S1; Fig. S2b). Gene Ontology (GO) and Kyoto Encyclopedia of Genes and Genomes (KEGG) pathway enrichment analyses (q-value <0.05) of the 1363 DEGs revealed the enrichment of diverse metabolic processes, including phenylpropanoid biosynthesis, flavonoid biosynthesis, ATP-binding cassette (ABC) transporters, and hormone synthesis and signal transduction (Fig. 3; Fig. S2c).

**Fig. 4:**
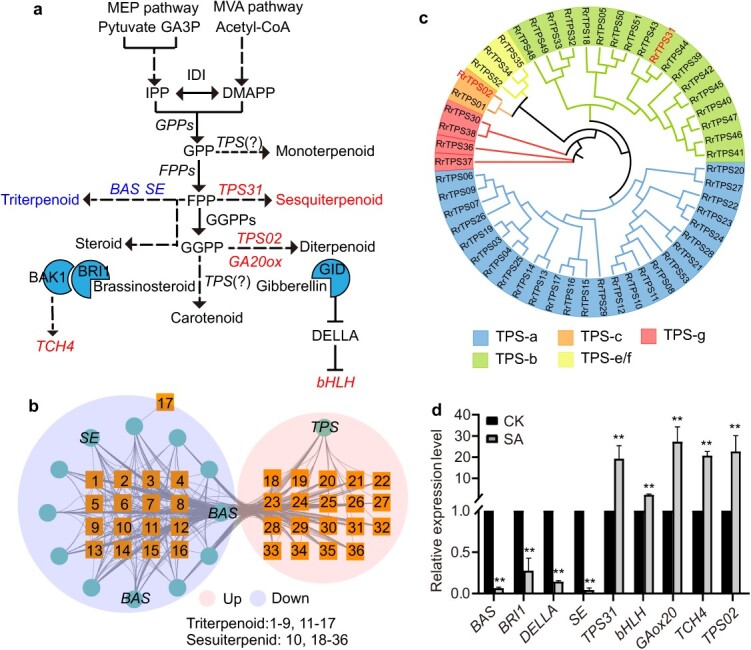
The synthesis of terpenoids and derivatives is influenced by aline–alkali stress in the rose petals. (a) Metabolites and DEGs in the terpenoid and derivative biosynthesis pathway. Upregulated and downregulated genes/metabolites are indicated in red and blue, respectively. (b) A correlation network of DEGs and metabolites involved in terpenoid biosynthesis. Blue circles indicate genes, and orange squares indicate metabolites. (c) Phylogenetic analysis of 53 rose TPS proteins*.* (d) qRT-PCR showing the expression levels of nine terpenoid-associated DEGs in the rose petals under saline–alkali stress. ^**^P < 0.01.

To establish a gene regulatory network responsive to saline–alkali stress in rose petals, we identified the coexpressed gene sets via Weighted Gene Co-expression Network Analysis (WGCNA), which has been widely used for identification of networks (modules) of highly correlated genes [24]. Based on the fragments per kilo base per million mapped (FPKM) expression matrix, 21 470 genes were clustered and divided into 38 modules decorated with diacritical colors (Fig. S3). Of the 38 modules, the blue and turquoise modules displayed significant correlations with saline–alkali responses in plant hormone signal transduction, plant–pathogen interaction, and Mitogen-Activated Protein Kinase (MAPK) signaling pathway (Table S2). In the blue and turquoise modules, some *EXPANSINs* (*EXPs*), *XYLOGLUCAN ENDOTRANSGLUCOSYLASEs/HYDROLASEs* (*XTHs*), *POLYGALACTURONASEs* (*PGs*), *PECTIN METHYL ESTERASEs* (*PMEs*), *PME INHIBITORs* (*PMEIs*), *PME INHIBITORs* (*PMEIs*), and *PECTATE LYASE* (*PL*) had altered expression levels after saline–alkali stress (Fig. 3b, c; Fig.S4). The homologs of these DEGs in other species are shown to function in cell wall reconstruction and stress resistance [25, 26]. The WGCNA results revealed that rose *PL* (evm.TU.Chr1.4007) and *CHS* (evm.TU.Chr3.237, a flavonoid structural gene) were the hub genes of blue and turquoise modules, respectively (Fig. 3b, c).

### Saline–alkali stress influences the biosynthesis of flavonoids in the rose petals

To examine how saline–alkali stress affects flavonoid synthesis in the rose petals, we selected 51 DEGs and 13 metabolites in the phenylpropanoid biosynthesis pathway to perform correlation tests. Pearson correlation coefficient showed that metabolite abundance and gene expression had strong correlation (R > 0.8 and *P* < 0.05) (Fig. S5a). The 47 DEGs and 13 metabolites were rearranged to corresponding positions in the phenylpropanoid pathway (Fig. S5b). Most compounds of flavones, flavonols, and flavanones and the core intermediate naringenin of flavonoid synthesis pathway showed higher abundances in petals of SA plants than in those of CK plants (Table S3). Consistent with this, the flavonoid structural genes *CHALCONE SYNTHASE* (*CHS*), *FLAVANONE 3-HYDROXYLASE* (*F3H*), *FLAVONOID 3′-HYDROXYLASE* (*F3′H*), *FLAVONOL SYNTHASE* (*FLS*), *DIHYDROFLAVONOL 4-REDUCTASE* (*DFR*), and *ANR* showed altered expression levels after saline–alkali stress (Fig. S5b). The qRT-PCR results validated that saline–alkali stress inhibited the expression of evm.TU.Chr3.237 (a *CHS* gene), evm.TU.Chr6.1383 (a *F3H* gene), evm.TU.Chr7.508 (a *F3’H* gene), and evm.TU.Chr5.6631 (a *ANR* gene) and increased the expression of evm.TU.Chr2.1593 (a *FLS* gene) (Fig. S5c). In addition, we found that 60% of non-flavonoid products in the phenylpropanoid pathway, including ferulic acid, 1-O-feruloylquinic acid, 5-O-p-coumaroylquinic acid, and caffeoyl shikimic acid, had fewer abundances in SA plants than in CK plants (Table S3). Consistently, the expression of the lignin-related genes *p-COUMARATE CoA LIGASE* (*4CL*), *CONIFERALDEHYDE 5-HYDROXYLASE (F5H)*, *HYDROXYCINNAMOYL TRANSFERASE* (*HCT*), *CAFFEIC ACID 3-O-METHYLTRANSFERASE* (*COMT*), *LACCASES* (*LAC*), *CINNAMOYL-CoA REDUCTASE* (*CCR*), and *CINNAMYL ALCOHOL DEHYDROGENASE* (*CAD*) was inhibited in rose petals by saline–alkali treatment (Fig. S5b). qRT-PCR confirmed lower expression levels of evm.TU.Chr7.2922 (a *4CL* gene), evm.TU.Chr2.4387 (a *F5H* gene), and evm.TU.Chr6.3700 (a *HCT* gene) in SA plants than in CK plants (Fig. S5c). Together, our results indicated that saline–alkali stress may impact different branches of the phenylpropanoid pathway in the rose petals.

### Saline–alkali stress influences the biosynthesis of terpenoids and derivatives in the rose petals

Terpenoids represent the most diverse class of chemicals produced by plants and are classified according to the number of five-carbon building blocks: monoterpenoids (C10), sesquiterpenoids (C15), diterpenoids (C20), sesterpenoids (C25), triterpenoids (C30), tetraterpenoids (C40), and polyterpenoids (C > 40, higher order terpenoids) [27]. To examine how saline–alkali stress affects terpenoid synthesis in rose petals, we analyzed 18 DEGs and 39 metabolites involved in the terpenoid biosynthesis pathway (Table S4). Pearson correlation coefficient of gene expression and metabolite abundance was high (R > 0.8 and *P* < 0.05). The 13 DEGs and 36 metabolites were positioned in the rose terpenoid biosynthesis pathway (Fig. 4a). Under saline–alkali stress, the abundances of 19 sesquiterpenoids were upregulated, while the abundances of 16 triterpenes and one sesquiterpenoid (aphanamol I) were downregulated (Fig. 4b). A significant correlation was observed between the abundances of the 19 sesquiterpenes and the expression of a *TPS* gene (evm.TU.Chr5.4711). This rose *TPS* gene was defined as *TPS31*, belonging to TPS-b subfamily, based on evolutionary relationships of 174 TPS proteins from rose (*R. rugosa*), Chinese rose (*Rosa chinensis*), and tea (*Camellia sinensis*) (Fig. 4c; Fig. S6). Most members of TPS-b subfamily are shown to be essential for the monoterpene, sesquiterpene, and isoprenoid synthesis [28]. By contrast, the abundances of the 16 triterpenoids were correlated with the expression of *BETA-AMYRIN SYNTHASE* (*BAS*, evm.TU.Chr6.4912) and *SQUALENE MONOOXYGENASE* (*SE*, evm.TU.Chr2.5861) (Fig. 4b).

**Fig. 5:**
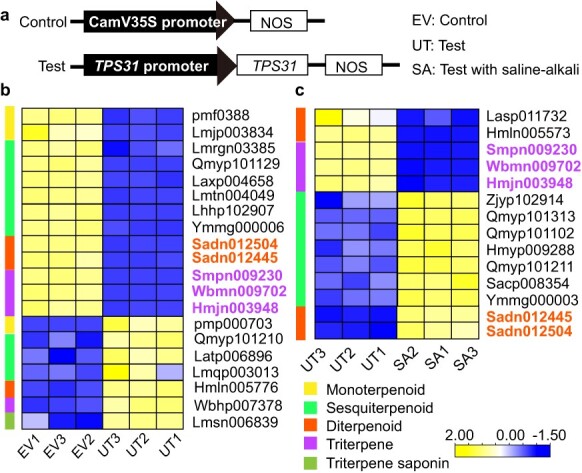
TPS31 functions in the terpenoid synthesis in response to saline–alkali stress. (a) The constructs for overexpression of empty vector (EV, control) or *TPS31* in tobacco. (b) Differentially accumulated terpenoids in *proTPS31::TPS31* plants compared with EV. (c) Differentially accumulated terpenoids in stressed (SA) *proTPS31::TPS31* plants compared to untreated (UT) plants. Different types of terpenoids are marked with different colors.

Isoprenoids serves as the precursors of several hormones including brassinosteroids (BRs), gibberellins (GAs), abscisic acid (ABA), and cytokinin (CTK) [29]. Our transcriptomic data revealed that saline–alkali stress caused significant changes in the expression of genes associated with BRs (*BRI1*, *BAK1,* and *TCH4*), GAs (*GA20ox*, *TPS*, *DELLA*, *GID1*, *bHLH*), ABA (*SIS*, *PYR/PYL*, and *PP2C*), and CTK (*CKX5* and *B-ARR*) in the rose petals (Fig. S7). It is possible that these hormones derived from isoprenoids participate in the regulation of plant defense against saline–alkali stress.

Nine genes (*BAS*, *BRI1*, *DELLA*, *SE*, *TPS31*, *bHLH*, *GA20ox*, *TCH4*, and *TPS02*) associated with terpenoid metabolism (Fig. S7) were selected for qRT-PCR analysis. The results were in agreement with the RNA-seq data (Fig. 4d). Together, our results indicated that saline–alkali stress may impact the biosynthesis of terpenoids and derivatives in the rose petals.

### Saline–alkali stress affects TPS31-mediated synthesis of terpenoids in tobacco

Since *TPS31* has a potential role in terpenoids synthesis in stressed rose petals (Fig. 4), this gene was selected or functional characterization. As expected, TPS31 protein sequence contains an RRX8W (R, arginine, W, tryptophan, and X, alternative amino acid) motif in the N-terminus and a DDXXD (D, aspartate) motif in the C-terminus (Fig. S8). Both motifs of TPS proteins are essential for catalysis of terpene cyclization [28]. We further generated transgenic tobacco overexpressing *proTPS31::TPS31* or empty vector (EV, the control) to determine the changes of terpenoid compounds under saline–alkali stress using UPLC-MS/MS. Three biological replicates of each treatment displayed similar expression profiles in PCA, indicating the effectiveness of data (Fig. S9). A total of 189 terpenoid compounds were detected in tobacco leaves with or without saline–alkali treatment (Fig. 5a). Compared with the controls, the abundances of 20 terpenoids, including monoterpenoid, sesquiterpenoid, ditepenoid, triterpen, terpene, and triterpene saponin, were significantly changed in *proTPS31::TSP31* plants (Fig. 5b; Table S5). Of them, 67% (2/3) of sesquiterpenes and all three triterpenes (arjunolic acid, asiatic acid, and madasiatic acid) had lower levels in *proTPS31::TSP31* plants than in EV plants (Table S5). These differentially accumulated sesquiterpenes contained aphaseic acid, nicosesquiterpene A, and costunolide, all which are used for repairing ischemic brain injury and as an anti-cancer agent [30, 31]. When *proTPS31::TSP31* plants suffered saline–alkali stress, the abundances of 14 terpenoids showed significant alterations compared with untreated plants. Of them, five sesquiterpenols (Zjyp102914, (1R,4aR,7R,8aR)-1,4a,8a-trimethyl-7-(prop-1-en-2-yl)-deca-hydronaphthalen-1-ol; Qmyp101313, Anhydro-β-rotunol; Qmyp101102, Arundinol B; Hmyp009288, epinootkatol and Ymmg000003, rosacorenol), two sesquiketones (Qmyp101211, Samboginone and Sacp008354, epishyobunone), and two cannabinoids (Sadn012445, cannabigerolic acid and Sadn012504, Δ-8-tetrahydrocannabinolic acid A) were upregulated, while three pentacyclic triterpenoids (Smpn009230, arjunolic acid; Wbmn009702, asiatic acid; and Hmjn003948, madasiatic acid) were downregulated (Fig. 5c). Consistently, the sesquiterpenes epinootkatol (Hmyp009288) and rosacorenol (Ymmg000003) in stressed rose petals had higher abundances than those in the untreated samples (Table S1). These results indicated that saline–alkali stress attenuated TPS31-inhibited accumulation of sesquiterpenes and enhanced TPS31-inhibited accumulation of pentacyclic triterpenoids in tobacco leaves. Furthermore, aline–alkali stress may promote the sesquiterpenes synthesis partially by the TPS31-dependent pathway.

**Fig. 6:**
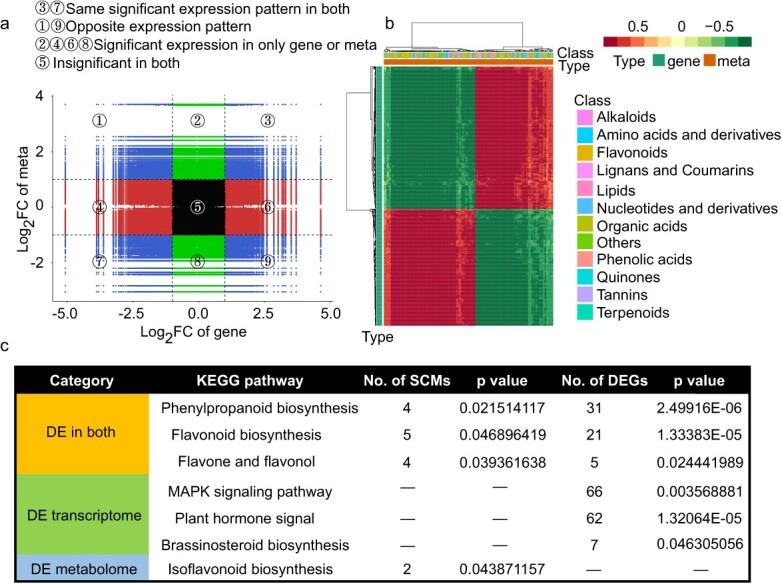
Conserved and differential regulation of metabolite accumulation and gene expression in the rose petals under saline–alkali stress. (a) Quadrant diagrams show the association of metabolites and genes. Black dotted lines represent different thresholds, and each point represents a gene or metabolite. ① − ⑨ quadrants are divided from left to right and from top to bottom. (b) Corheatmap of DAMs and DEGs. (c) KEGG pathway (*P <* 0.05) was enriched in the group of metabolic pathways, that were significantly changed at both the mRNA and metabolic levels or significantly changed at the mRNA or metabolic level.

### Integrative analysis of differentially accumulated metabolites and differentially expressed genes

To understand the regulatory mechanism of metabolite synthesis under saline–alkali stress in the rose petals, 17 735 genes and 1815 metabolites identified here were subjected to correlation tests. The expression levels of 14 697 genes were correlated with the abundances of 764 metabolites, with R (Spearman) > 0.8 and *P-*value <0.05. Nine quadrant maps illustrated the correlative relationships of these genes and metabolites (Fig. 6a). Genes and metabolites located in the third and seventh quadrants showed identical expression patterns, implying a positive association between them. A total of 9.53% of DEGs and DAMs showed the alterations at both the transcriptional and metabolic levels, and 90.47% showed discordant alterations (Fig. 6b). DEGs/DAMs at both the mRNA and metabolic levels were enriched in processes including phenylpropanoid biosynthesis, flavonoid biosynthesis, and flavone and flavonol biosynthesis (Fig. 6c). DEGs specifically at the mRNA level were involved in MAPK signaling pathway, auxin and GA signal transduction, and BR biosynthesis. DAMs specifically at the metabolic level were involved in isoflavonoid biosynthesis.

**Fig. 7:**
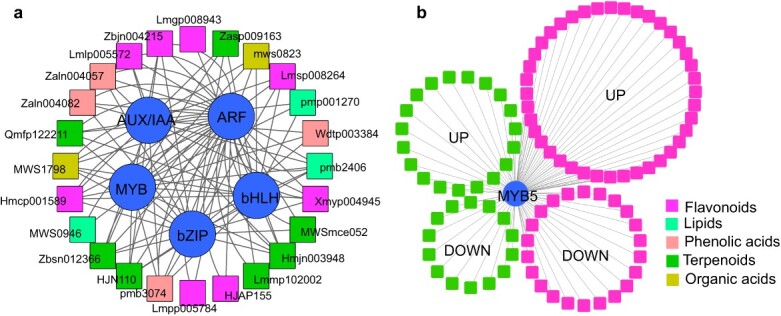
The correlation of DAMs and DETFs in stressed rose petals. (a) Correlation analysis of the top 50 DAMs and DETFs. (b) The correlation of MYB5 and differentially accumulated flavonoids and terpenoids.

To identify saline–alkali-responsive TFs associated with flavonoids and terpenoids, top 50 differentially expressed TFs (DETFs) and 50 DAMs at both the mRNA and metabolic levels were selected for analysis of Pearson correlation coefficients. As shown in Fig. 7a, the members of *MYB*, *ARF*, *AUX/IAA*, *bZIP*, and *bHLH* gene families showed high correlations with various types of metabolites in stressed rose petals. Of them, MYB5 (evm.TU.Chr3.3297) was correlated with 62 flavonoids and 33 terpenoids (Fig. 7b). This was consistent with previous studies that rose MYB5 and its homolog MYB23 in *A. annua* regulate PA and artemisinin synthesis [18, 19]. In this network, saline–alkali stress caused ~20% reductions in the abundances of two PAs (procyanidin B3, pme0436 and procyanidin B2 3’-O-Gallate, Wcfn003698), and >3-fold increases in the abundances of multiple sesquiterpenoids, including rosacorenol (Ymmg000003), 5α,6α,7β,10β-11α,13-dihydro-4(15)-eudesmene-12,6-olide tsoongiodendroonolide (Lhhp102908), and shizukanolide A (Lmcp006589) (Table S1).

### MYB5 directly suppresses *TPS31* expression in response to saline–alkali stress

**Fig. 8:**
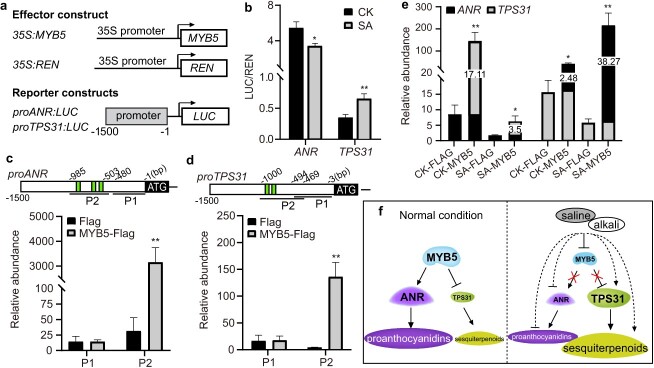
Saline–alkali stress attenuates transcriptional regulation of *ANS* and *TPS31* expression by MYB5. (a, b) The effector and reporter constructs used for transient expression assays in poplar leaf protoplasts (a). Luciferase/Renilla (LUC/REN) ratio in the protoplasts cotransformed with *35S::MYB5/35S::REN* and *proANR::LUC* or *proTPS31::LUC* with or without saline–alkali treatment. *REN* expression was used as an internal control. Data are the means ± SD of three biological replicates. (c, d) ChIP-qPCR showing the specific binding of MYB5 to P2 in the *TPS31* and *ANR* promoters in rose petal protoplasts. An MYB-binding site (MRE) was marked with a green box. (e) ChIP-qPCR showing saline–alkali treatment affect the bindings of MYB5 to P2 in the *TPS31* and *ANR* promoters in the protoplasts. Data represent mean ± SD of three biological replicates. (f) A model of the MYB5-ANR/TPS31 module transcriptionally regulating proanthocyanidins and sesquiterpenoids accumulation in the rose petals in response to saline–alkali stress. Student’s *t-*test, ^*^P < 0.05; ^**^P < 0.01.

Integrative analysis of DETFs and DAMs exhibited a high correlation between *MYB5* expression and flavonoid/terpenoid accumulation in stressed rose petals (Fig. 7a). This finding promoted us to examine whether MYB5 participates in TPS31-mediated synthesis of terpenoids under saline–alkali stress. Transient expression assays revealed that MYB5 activated *ANR* expression in poplar leaf protoplasts (Fig. 8a, b), which was consistent with previous study that overexpression of *MYB5* promoted *ANR* expression and PA accumulation in rose and tobacco [18]. Using this system, we found that MYB5 suppressed *TPS31* expression in poplar leaf protoplasts (Fig. 8a, b). Saline–alkali stress attenuated the activation of *ANR* and suppression of *TPS31* by MYB5. Consistent with these, *MYB5* and *ANR* showed similar responses to saline–alkali stress, but *MYB5* and *TPS31* had opposite responses in stressed rose petals (Fig. 4d; Fig. S5c,10). The 1500-bp *ANR* and *TPS31* promoter fragments contain several MYB Responsive Elements (MREs, TTATC, and GAA/TTC [32],) predicted by PlantPAN (http://plantpan.itps.ncku.edu.tw/plantpan4/) (Fig. 8c, d). This finding suggests that these MREs in the *ANR* and *TPS31* promoters may be recognized by MYB5. Chromatin immunoprecipitation (ChIP)-qPCR results revealed that MYB5 was significantly enriched at P2 covering MREs but not at P1 (the negative controls) of *ANR* or *TPS31* promoter in rose petal protoplasts (Fig. 8c, d). After saline–alkali stress, MYB5 abundances decreased from 17.11 to 3.5-fold at P2 of the *ANR* promoter, and increased from 2.48 to 38.27-fold at P2 of the *TPS31* promoter in protoplasts expressing *MYB5-FLAG* relative to the controls (Fig. 8e). All these results indicated that saline–alkali stress may inhibit MYB5-mediated regulation of *ANR* and *TPS31* expression.

## Discussion

### Saline–alkali stress influences MYB5-mediated regulation of proanthocyanidin and sesquiterpenoid synthesis

Plants synthesize >170 000 terpenoids with a variety of biological functions. TPSs are responsible for the final steps in terpenoid synthesis [28]. Currently, *TPS* gene families have been identified in various species, including *Arabidopsis thaliana* (33 *TPS* members) [33], *Vitis vinifera* (69 *TPSs*) [34], *Eucalyptus globulus* (106 *TPSs*) [35], and *R. chinensis* (49 *TPSs*) [28]. The members of TPS-b subfamily are implicated in monoterpene, sesquiterpene, and isoprenoid synthesis [28]. For instance, in Arabidopsis inflorescence, MYC2 integrates GA and JA signals into transcriptional regulation of *TPS21* and *TPS11*, promoting the synthesis of sesquiterpenes, especially (E)-β-caryophyllene [36]. In peach, PpERF61 activates the expression of *PpTPS1* and *PpTPS3*, promoting the synthesis of the monoterpene linalool during fruit ripening [37]. In tea plant, *CsTPS08* and *CsTPS10* participate in the regulation of pest defense and aroma quality [38]. Here, integrative analysis of metabolome and transcriptome revealed a high correlation between 19 sesquiterpenes and *TPS31*, a member of rose TPS-b subfamily. Ectopic expression of *TPS31* in tobacco driven by its own promoter resulted in significant alterations in the abundances of 20 terpenoids, most of which are therapeutically valuable [39]. Our metabolic data confirmed the previous speculation that overexpression of a single *TPS* produces different terpenes in planta, despite being validated *in vitro* for just one specific compound [15].

In plants, several MYB proteins play key roles in controlling both flavonoid and terpenoid biosynthesis. In tomato, overexpression of *SlMYB75* increases the levels of anthocyanin and aroma volatiles (aldehyde, phenylpropanoid-derived, and terpene volatiles), but reduces sesquiterpene levels through direct suppression of *SlTPS12*, *SlTPS31*, and *SlTPS35* expression [16, 17]. In variegated grape berries, MYB24 orchestrates monoterpene and flavonol metabolism through direct activation of the expression of monoterpene-associated *TPS35/09* and flavonol-associated *HY5 HOMOLOGUE* (*HYH*) as light responses to anthocyanin depletion [15]. Here, we found that flavonoid/terpenoid abundances had a high correlation with *MYB5* expression in stressed rose petals, which was consistent with several previous studies. MYB5 regulates the anthocyanin and PA synthesis in response to wounding and oxidation [18]. Heterologous expression of grape *MYB5b* in tomato inhibits phenylpropanoid metabolism and increases the amount of beta-carotene in fruits [40]. MYB23, the homolog of MYB5 in *A.annua*, regulates artemisinin synthesis [19]. These findings support a hypothesis that MYB5 may promote PA accumulation via *ANR* and inhibit sesquiterpene accumulation via *TPS31* (Fig. 8f, left). Saline–alkali stress strongly suppressed *MYB5* transcription in salt-sensitive rose variety ‘Fenghua’, correlating with decreased PA and anthocyanin levels in rose under salt stress [41]. Decreasing *MYB5* removes its activation of *ANR* expression, inhibiting PA accumulation, and also removes its suppression of *TPS31* expression, promoting sesquiterpene accumulation after saline–alkali stress (Fig. 8f, right). It is noted that stress removes the suppression of *TPS31* expression by MYB5, which was inconsistent with stress-induced increase in MYB5 binding to MBRs in *TPS31* promoter. A possible reason is that MYB5 relies on its interacting partners to inhibit *TPS31* expression. Extensive studies have shown that MYB, bHLH, and WD40 proteins form a complex to regulate flavonoid and terpenoid biosynthesis [42].

### Saline–alkali stress causes the redirection of major metabolic flux in the rose petals

The petals of rose are widely used in the food industry, perfumery, and cosmetics, due to richness of beneficial secondary metabolites [5]. Here, we found that saline–alkali stress caused transcriptional and metabolic changes in rose petals at full opening stage. Of the altered metabolites, flavonoids and terpenoids accounted for the largest proportions. Similarly, under saline–alkali stress flavonoid abundances were also changed in apple [43] and oil seed rape [44]. It is well known that flavonoids prevent the accumulation of reactive oxygen species (ROS) in plants [45]. Thus, in stressed rose petals, increased flavonoids may facilitate reducing the damage caused by stress. In the phenylpropanoid biosynthesis pathway, 60% of non-flavonoid products showed reduced abundances after saline–alkali stress (Fig. 5; Table S3). Consistently, the expression of the lignin biosynthetic genes *4CL*, *F5H*, *HCT*, *COMT*, *PRX*, *LAC*, *CCR*, and *CAD* was downregulated under saline–alkali stress (Fig. 5). Combined with previous studies in alfalfa [46] and apple [43], we speculated that the inhibition of lignin synthesis in stressed rose petals may be helpful for plants to adapt to this stress. In addition, we found that saline–alkali stress inhibited triterpene accumulation in rose petals. This was consistent with a recent report that the abundances of triterpenoids, such as amyrins and betulinic acid, were reduced in the roots of *Sesuvium portulacastrum* when exposed to salinity [47]. All these results indicated that altered metabolic flux is an important defense strategy of rose against saline–alkali stress.

### Saline–alkali stress influences cell wall reconstruction in the rose petals

The plant cell walls are directly exposed to the environment and are critical for resetting the balance between growth and the stress response [2]. To survive in stressed soils, plants have evolved into the complex dynamic architectures of primary cell walls that mainly composed of cellulose, hemicellulose, and pectin. Cell wall loosening requires the acidification of the cell walls; protons displace Ca ^2+^ ions from their linkage positions between pectin molecules [25]. Extensive studies in multiple plants show that salt stress results in significant alterations in the expression of genes associated with cell wall reconstruction, including *EXP*, *XTH*, *PG*, *PL*, *PME*, and *PMEI*. In maize, *ZmEXPA1*, *ZmEXPA3*, *ZmEXPA5*, *ZmEXPB1*, and *ZmEXPB2* are required for cell enlargement, leading to a reduction of the ionic toxicity induced by salinity [48]. In tobacco, overexpression of wheat *TaEXPA2* improves salt tolerance by regulating Na^+^/K^+^and antioxidant competence [49]. In rice, mutation of *PME*/*OsTSD2* inhibits salt-induced expression of genes associated with ion homeostasis (*OsKAT1*, *OsSOS1*, and *OsHKT1*), causing an increase of Na^+^ level and a reduction of K^+^ level in shoots [50]. Upregulated or downregulated expression of *XTHs* from poplar, *Medicago truncatula*, and *Salicornia europaea* affects the abundance of xyloglucan and produces larger and more irregular cells with a higher density of cell walls and intercellular spaces, enhancing plant tolerance to salt stress [51–53]. Our transcriptome data showed that most of *EXP*, *XTH*, *PG*, *PL*, *PME*, and *PMEI* genes were inhibited by saline–alkali stress in rose petals (Fig. S4). Of them, the *PL* gene evm.TU.Chr1.4007 was identified as a hub of the network (Fig. 3). It is possible that these *EXP*, *XTH*, *PG*, *PL*, *PME*, and *PMEI* genes contribute to cell wall loosening of rose petals, enhancing plant tolerance to saline–alkali stress.

Plants have evolved cell wall integrity signaling pathways to maintain cell wall homeostasis in response to environmental stresses [2]. In Arabidopsis, the plasma membrane-localized receptor-like protein kinase FERONIA (FER) senses salinity-mediated softening of the cell walls by directly interacting with pectins [54]. The RAPID ALKALINIZATION FACTOR (RALF) peptides RALF22/23 are physically associated with cell wall leucine-rich repeat extensins (LRX) 3/4/5 and FER. Salt stress causes the S1P protease-dependent release of mature RALF22 peptides, which in turn induces the internalization of FER via an endosomal pathway [55]. In our transcriptome data, the expression levels of rose *FER*, *LRX8*, and *RALF10* were upregulated under saline–alkali stress in petals (Table S1). This result, combined with the finding that Arabidopsis LRX8 interacts with RALF4/19 in the cell walls to regulate pollen tube growth [56], supported a hypothesis that the LRX8-RALF10-FER module may function in salt-induced cell wall damage of rose petals. In addition, we found that ABA signaling was activated in stressed rose petals (Fig. S5). It is known that there are extensive cross talks between ABA signaling and FER signaling [57]. Thus, the LRX8-RALF10-FER module may coordinate petal expansion and salt stress response. Further studies need be carried out to illuminate these ambiguities.

## Materials and methods

### Plant materials and salt–alkali treatment

The *R. rugosa* variety ‘Fenghua’, a salt-sensitive and edible cultivar planted widely in China [58], was used in this study. Plants were grown in a greenhouse with long-day conditions (16-h light/8-h dark) at 24 ± 1°C. For salt and alkali treatment, 1-year-old rose plants were watered with the solution containing 0.4% saline (NaCl, Na_2_SO_4_, and NaHCO_3_) and alkali (pH 8.0) for 1 week. One-month-old tobacco (*Nicotiana benthamiana*) plants expressing empty vector (EV) or *proTPS31::TPS31* were treated with the salt–alkali solution for 4 days. The control plants remained in water.

### Metabolomic analysis

The rose petals at full opening stage were collected for a widely targeted metabolome analysis by Metware Biotechnology (Wuhan, China). The tobacco leaves expressing EV or *proTPS31::TPS31* were collected for detection of terpenoid compounds. For each treatment, three independent biological replicates were set. One hundred milligrams of powdered samples were dissolved in 1 ml 70% aqueous methanol and extracted overnight at 4°C. After centrifugation at 10 000 g for 10 min, the supernatants were filtrated using a 0.22-μm filter membrane (SCAA-104, ANPEL, Shanghai, China) for UPLC-tandem mass spectrometry analysis (UPLC-ESI-MS/MS system; UPLC: Nexera X2, Shimadzu, Kyoto, Japan; MS: Applied Biosystems 4500 QTRAP, Applied Biosystems, Waltham, MA, USA). Metabolic profiling was analyzed using Analyst 1.6.3 software (AB Sciex, Darmstadt, Germany). According to the guidelines by Fernie *et al*. 2011 [59], the parameters for metabolite identification were provided in Supplemental Table S1. The metabolites were quantified using the MRM mode. DAMs were evaluated using the variable importance in projection (VIP) scores (VIP ≥ 1; log_2_ fold change (FC) is ≥1 or ≤−1) via the orthogonal partial least squares discriminant analysis (OPLS-DA). OPLS-DA was generated using R package MetaboAnalystR. The data were log-transformed (log2) and mean-centered prior to OPLS-DA analysis. A permutation test (200 permutations) was carried out to avoid overfitting. The raw metabolome data is available in the MetaboLights (MTBLS10591).

### RNA-seq

Total RNAs were isolated from rose petals used in metabolome analysis. Library construction, RNA sequencing, identification of DEGs, and functional annotation were performed following our previous study [60]. RNAs were sequenced on Illumina HiSeq4000 (6G 150bp paired-end reads). Sequence data with base-pair qualities in the Q ≥ 20 were extracted by custom Perlscripts. The filtered reads were mapped to the rose genome (http://eplantftp.njau.edu.cn/Rosa_rugosa/Rosa_rugosa_genome.fasta) using TopHat2 software with default parameters. FPKM was used for quantification of gene/transcription levels. DEGs were determined using the edgeR package (http://www.bioconductor.org/packages/release/bioc/html/edgeR.-html) based on the following criteria: (1) FPKM >1; (2) false discovery rate (FDR) < 0.05; (3) Log_2_FC is ≥1 or ≤−1. To infer the putative functions of the DEGs, Gene Ontology (GO) and Kyoto Encyclopedia of Genes and Genomes (KEGG) enrichment analyses were conducted using the GOseq R package and KOBAS (v2.0) software, respectively. GO terms with adjusted *P* < 0.05 were considered significantly enriched. The raw transcriptome data is available in the NCBI SRA (SUB14574392).

### Weighted gene coexpression network analysis

Gene coexpression modules with distinct expression patterns were identified using the weighted gene coexpression network analysis (WGCNA) package [24]. A total of 21 470 genes were selected for module construction. WGCNA depends on the soft-thresholding power b = 17, with fitting curve being close to 0.8. The minimum number of genes was set as 50 to ensure high reliability of the results. The parameter of modules merging is 0.25. In a certain module, hub genes were filtered based on maximum significance with genotype and high module membership by the chooseTopHubInEachModule package. The genetic network map was created using the Cytoscape software.

### Integrative analysis of metabolomic and transcriptomic data

DEGs and DAMs in the same group were mapped to KEGG pathways for integrative analysis. Nine-quadrant graphs and correlation scatter plots were used to show the genes and metabolites with high (>0.8) Pearson correlation coefficients (*P-*value <0.05).

### Transient gene expression assays

The coding region of MYB5 was amplified from rose cDNA with gene-specific primers (Table S7) and ligated between the 35S promoter and the nopaline synthase (NOS) terminator in the modified pBI221 vector to create the effector construct. *REN* expression was used as an internal control. The 1500-bp promoter fragments of *ANR* and *TPS31* genes were individually ligated upstream of pGreenII0800-LUC to create the reporter constructs. Protoplasts were isolated from full-opening petals of rose and the leaves of poplar (*Populus alba* × *Populus glandulosa* ‘84 K’) following the method as described [61]. These protoplasts were transfected with different combinations of reporter and effector. For detecting the effects of saline–alkali stress on MYB5 regulation of *ANR* and *TPS31,* the protoplasts were pretreated with 0.4% salinity solution (pH = 8.0) for 0.5 h. LUC luminescence and REN fluorescence were measured by a luminometer (Promega) with a dual-luciferase reporter assay kit (Vazyme, Nanjing, China). Relative ratio of LUC activity to REN activity represents the expression levels of the reporter genes. Data are the means of five independent samples from three biological replications.

### qRT-PCR

Total RNAs were isolated using a Steady Pure Plant RNA Extraction Kit (Accurate Biotechnol, Changsha, China) and reverse-transcribed into cDNA using an Evo M-MLV RT Mix Kit (Accurate Biotechnol, Changsha, China). Reactions were carried out on a QuantStudio 1 Real-Time PCR system (Thermo Fisher Scientific, Waltham, USA) with the SYBR® Green Premix Pro Taq HS qPCR Kit (Accurate Biotechnol, Changsha, China). For each sample, three experiments were conducted independently. *RrGAPDH* (evm.TU.Chr1.3219) was used as the reference gene. The expression levels were calculated following the 2^−ΔΔCT^ method [62].

### ChIP-qPCR

The coding region of *MYB5* was cloned into pUC19-35S-FLAG-35S-sGFP. The resulting construct was transfected into the protoplasts of rose petals. ChIP assays were performed in 5 × 10^6^ protoplasts with an anti-FLAG antibody as described previously [60]. One-fiftieth of the supernatants before adding antibodies were used as input. qPCR was conducted on a Quant Studio 1 Real-Time PCR systemusing the SYBR® Green Premix Pro Taq HS qPCR Kit. *RrGAPDH* was used as the reference gene.

### Phylogenetic analysis

Full-length TPS protein sequences were identified in the genomes of *R. rugosa*, *R.* chinensis, and *C. sinensis* using TBtools [63]. A phylogenetic tree was generated using the neighbor-joining (NJ) method in MEGA 7.0. Bootstrap analysis with 1000 replicates was used to evaluate the significance of the nodes.

## Supplementary Material

Web_Material_uhae243

## Data Availability

The data and materials supporting the conclusions of this study are included in supplementary information.
