# Supplementary material for: Saline-alkali stress affects the accumulation of proanthocyanidins and sesquiterpenoids via the MYB5-ANR/TPS31 cascades in the rose petals
Source: Hortic Res. 2024 Aug 30;11(11):uhae243. doi: 10.1093/hr/uhae243 (PMC11554761; doi:10.1093/hr/uhae243)
Supplement: Web_Material_uhae243 [file web_material_uhae243.zip › Supplemental datas/Supplemental Figures 1-9.docx]

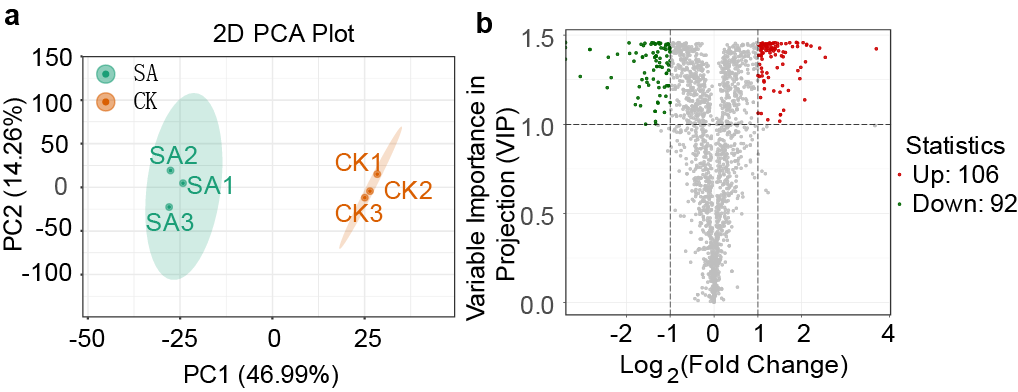


**Supplemental Figure 1.** Metabolome analysis of the rose petals under saline-alkali stress (a) PCA analysis of the abundances of metabolites in treated (SA) and control (CK) groups. (b) Volcano plot of the metabolites between control and treated groups.


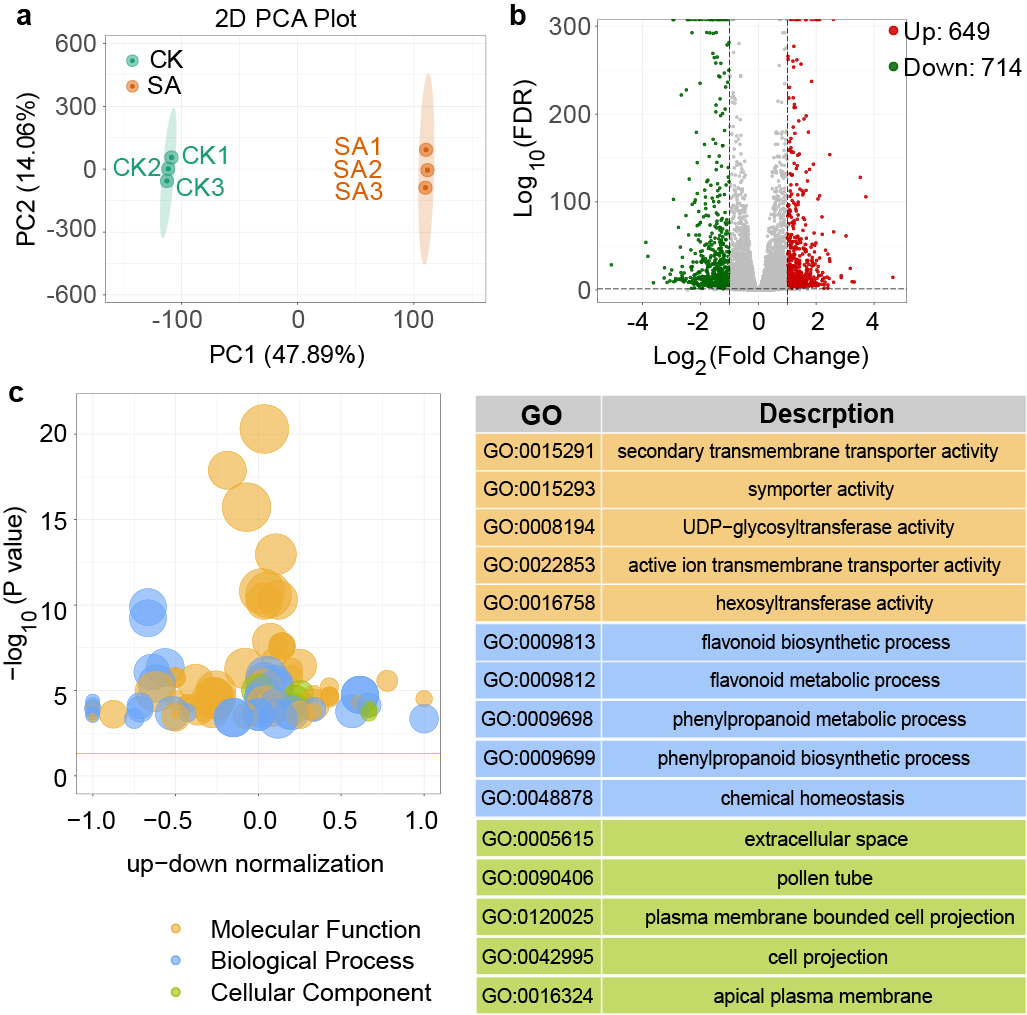


**Supplemental Figure2.** Transcriptome analysis of the rose petals under saline-alkali stress. (a) PCA analysis of the expression of unigenes in treated (SA) and control (CK) groups. (b) Volcano plot of the genes between control and treated groups. (c) GO functional classification of differentially expressed genes.


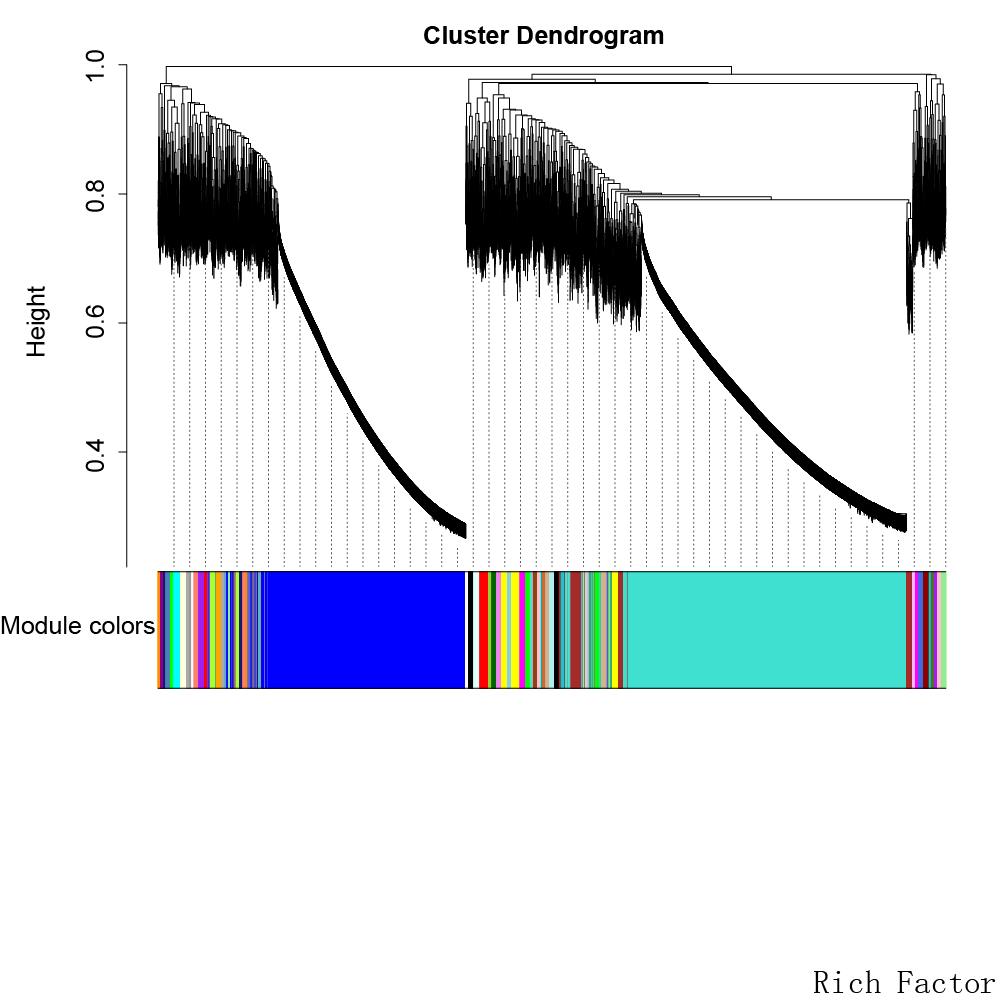


**Supplemental Figure 3.** Hierarchical dendrogram showing the co-expression modules identified by WGCNA. A total of 38 modules were identified based on calculation of eigengenes and each module was decorated with a different color.


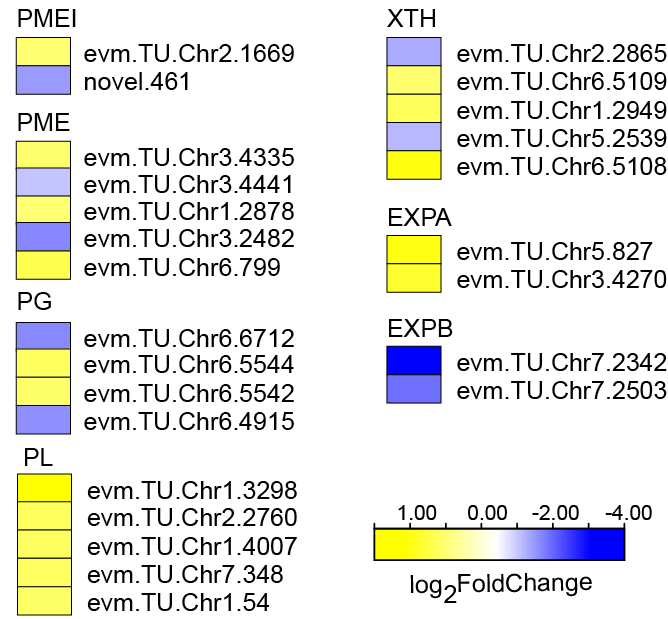


**Supplemental Figure 4.** Differentially expressed genes associated with cell expansion in stressed rose petals.


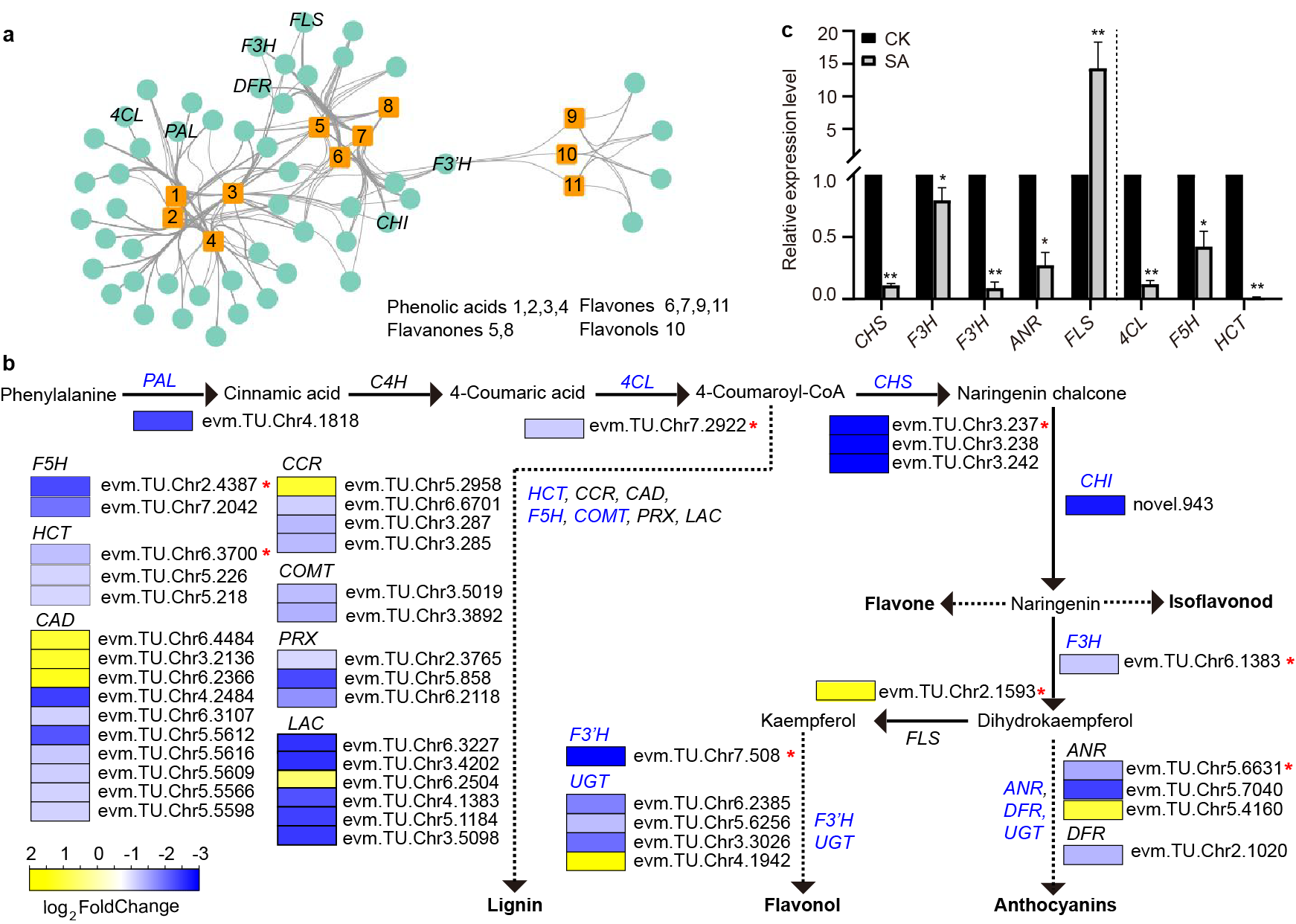


**Supplemental Figure 5.** Flavonoid synthesis is influenced by saline-alkali stress in the rose petals. (a) Correlation network of the expression of DEGs and the abundances of metabolites in phenylpropanoid metabolism. Blue circles represent genes, and orange squares represent metabolites. (b) DEGs and metabolites in the phenylpropanoid biosynthesis pathway. (c) qRT–PCR showing the expression levels of 7 DEGs associated with flavonoid and lignin in the rose petals under saline−alkali stress. These genes are marked with red stars in (b). ^*^*P* < 0.05; ^**^*P* < 0.01.


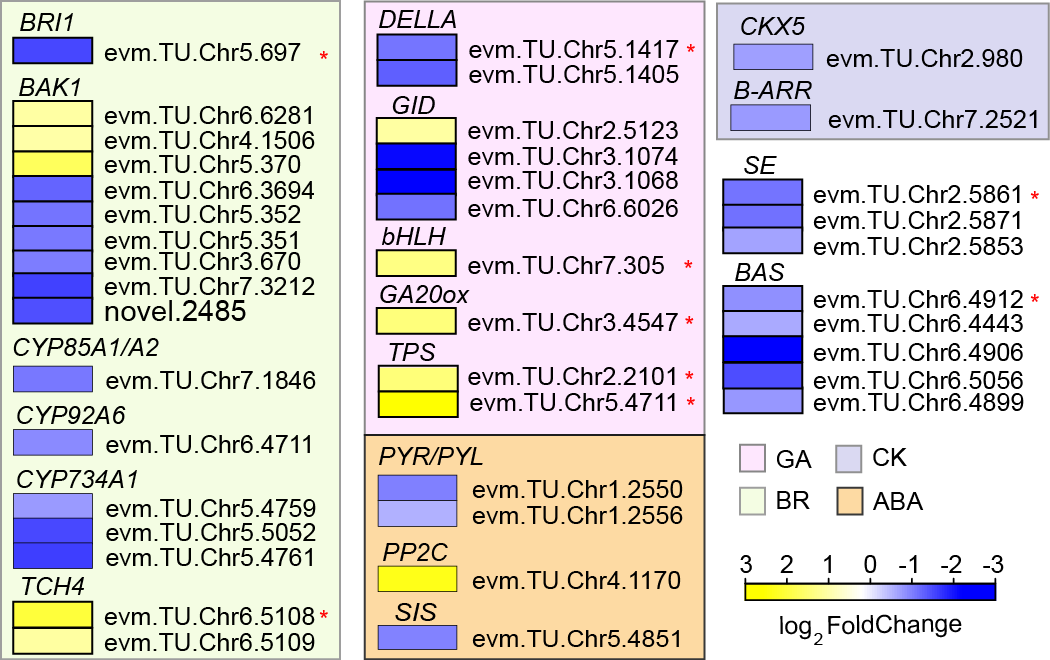


**Supplemental Figure 6.** Differentially expressed genes associated with hormone synthesis and signal transduction. Heat map of the transcripts of DEGs associated with hormones synthesis and signal transduction. Genes selected for qRT-PCR validation are marked with red stars.

**
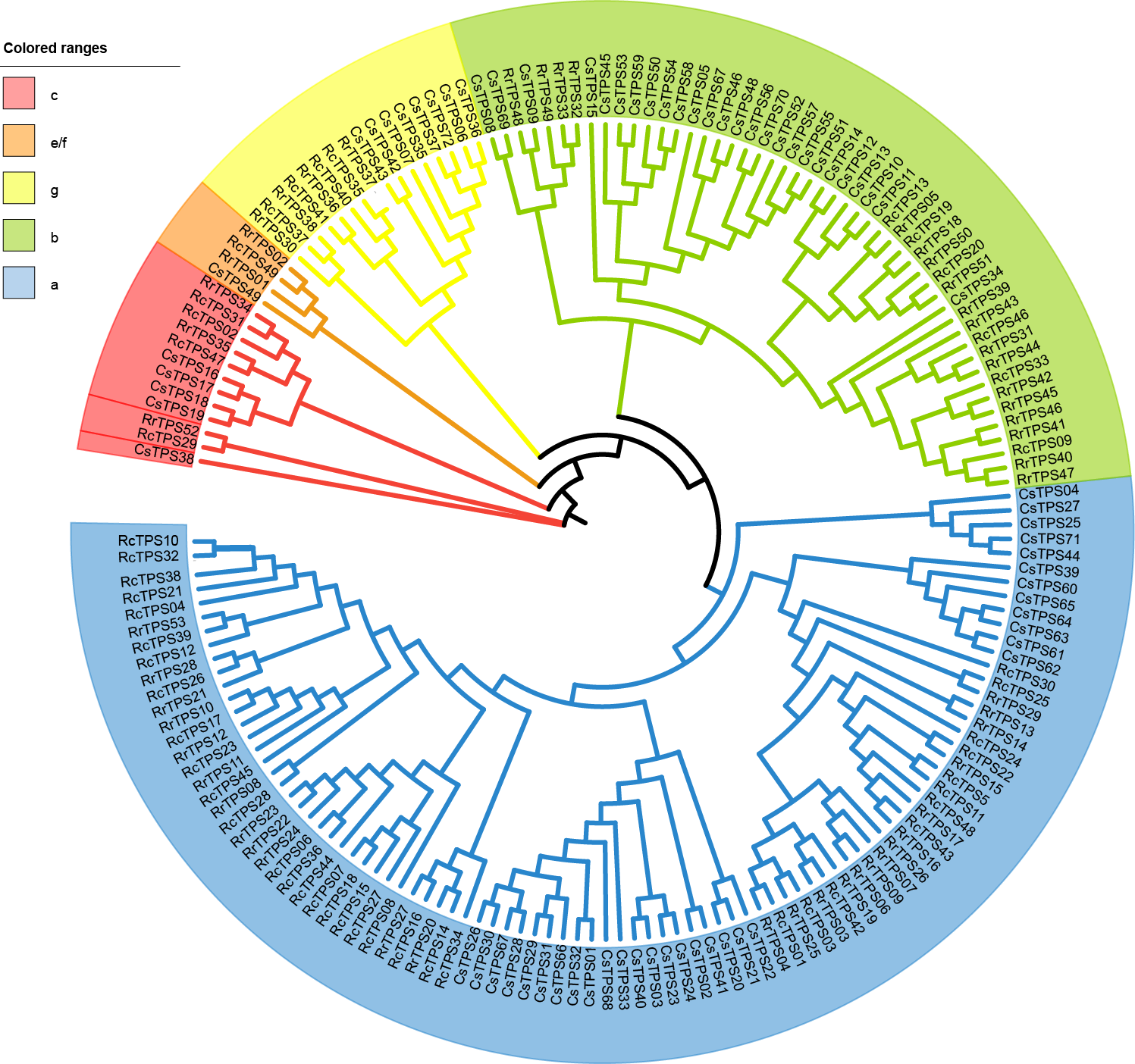
**

**Supplemental Figure 7.** Phylogenetic analysis of TPS proteins from *Rosa rugosa, Rosa chinensis* and *Camellia sinensis*. Different subfamilies are marked with different colors.

**
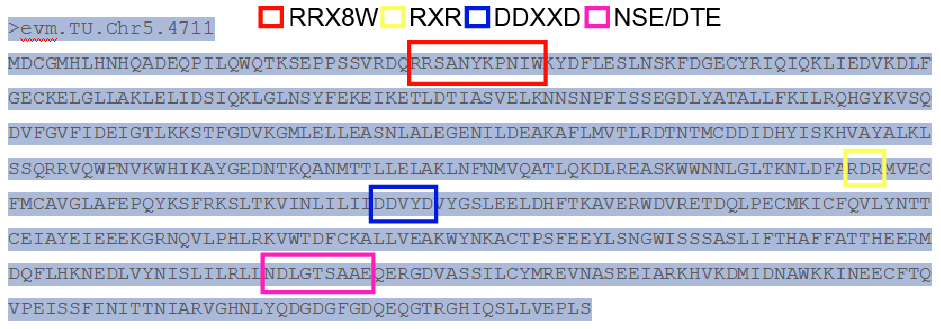
**

**Supplemental Figure 8.** The conserved domains of rose TPS31 protein sequence.


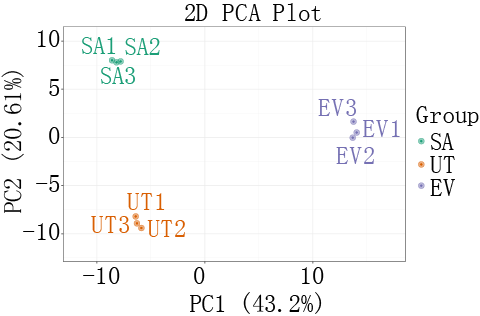


**Supplemental Figure 9.** PCA analysis of the abundances of terpenoids in tobacco leaves expressing *proTPS31::TPS31* or empty vector (EV, the control) with (SA) or without (UT) saline-alkali treatment.
